# RNAexinv: An extended inverse RNA folding from shape and physical attributes to sequences

**DOI:** 10.1186/1471-2105-12-319

**Published:** 2011-08-03

**Authors:** Assaf Avihoo, Alexander Churkin, Danny Barash

**Affiliations:** 1 Department of Computer Science, Ben-Gurion University, 84105 Beer Sheva, Israel

## Abstract

**Background:**

RNAexinv is an interactive java application that performs RNA sequence design, constrained to yield a specific RNA shape and physical attributes. It is an extended inverse RNA folding program with the rationale behind that the generated sequences should not only fold into a desired structure, but they should also exhibit favorable attributes such as thermodynamic stability and mutational robustness. RNAexinv considers not only the secondary structure in order to design sequences, but also the mutational robustness and the minimum free energy. The sequences that are generated may not fully conform with the given RNA secondary structure, but they will strictly conform with the RNA shape of the given secondary structure and thereby take into consideration the recommended values of thermodynamic stability and mutational robustness that are provided.

**Results:**

The output consists of designed sequences that are generated by the proposed method. Selecting a sequence displays the secondary structure drawings of the target and the predicted fold of the sequence, including some basic information about the desired and achieved thermodynamic stability and mutational robustness. RNAexinv can be used successfully without prior experience, simply specifying an initial RNA secondary structure in dot-bracket notation and numerical values for the desired neutrality and minimum free energy. The package runs under LINUX operating system. Secondary structure predictions are performed using the Vienna RNA package.

**Conclusions:**

RNAexinv is a user friendly tool that can be used for RNA sequence design. It is especially useful in cases where a functional stem-loop structure of a natural sequence should be strictly kept in the designed sequences but a distant motif in the rest of the structure may contain one more or less nucleotide at the expense of another, as long as the global shape is preserved. This allows the insertion of physical observables as constraints. RNAexinv is available at http://www.cs.bgu.ac.il/~RNAexinv.

## Background

RNAexinv is a user friendly computer tool that extends the inverse RNA folding problem to include physical attributes. Before elaborating on the inverse problem, one should begin by mentioning the classical RNA folding problem that aims to predict the secondary structure of a given RNA sequence. Software packages are nowadays available that contain RNA thermodynamic parameters [1-3] and predict the secondary structure from sequence by energy minimization. The inverse RNA folding problem was introduced in [4,5] and as its name suggests, it aims to design a sequence that folds into a given RNA secondary structure. A brute force approach that searches all the possible sequences is not a viable option because the number of sequences grows exponentially as κ^n^, where κ is the number of letters in the alphabet (κ = 4 for RNAs) and n is the length of the sequence [4]. Therefore, starting from RNAinverse that is available in the Vienna RNA package [5], various other methods [6,7] that do not compute the whole solution space were developed for the inverse RNA folding problem. It should be noted that both RNAinverse [5] and INFO-RNA [7] contain a p-mode option, where the objective function is including the probability that the target structure forms, thus considering thermodynamic stability. Recently, inspired by the physical aspects of RNA secondary structure [8], an extended inverse RNA folding problem was suggested [9], which adds several non-structural constraints to the desired output in conjunction such as thermodynamic stability and mutational robustness. This extension may help incorporate some important properties of natural RNAs to the design problem. For example, in recent years, several developed methodologies that were meant to address RNA secondary structure mutational analysis (e.g., [10,11]) mention the potential importance of mutational stability to RNA design.

Here we describe the software implementation of a new method for the extended inverse RNA problem. Unlike the method in [9] that utilizes parallel evolutionary computation and is relatively expensive, the method described here is closer in type to [5-7] and significantly reduces the computation time relative to [9]. It comprises two phases, the first of which is to identify a good initial candidate whose folding closely resembles that of the desired structure. The second phase is a simulated annealing heuristic with a four-nucleotide look ahead local search function. The first step in our method is essentially RNAinverse from the Vienna RNA package to obtain a good, initial sequence whose folding approximates that of the desired structure. Then the RNAinverse result is used as the starting sequence in the local search for the desired sequence of the extended problem. Search goals are defined not only by the desired structure, but also by the thermodynamic stability and mutational robustness parameters. The end result is a sequence with a greater number of natural qualities than exhibited by a random sequence with the desired structure. The method employed is described in more detail in the next section.

## Implementation

### Method

We now elaborate in more detail on the various stages that the method consists of, starting from the first. For our requirements, using RNAinverse with a random start point is preferable over more "fixed" inverse RNA folding strategies such as INFO-RNA [7]. In a diversion from the RNAinverse approach, which uses a random start point, INFO-RNA has a deterministic first stage and only a stochastic local search [12] during the second stage. A random start point is preferable in our specific case since it will produce different start sequences for our extended search rather than a fixed starting point, which could cause all the starting points to resemble each other.

Starting with the sequence that was generated by RNAinverse from the Vienna RNA package with a random start point, we search for local optima using iterative mutating. Neighboring sequences are assessed according to an objective function that takes into account all the parameters: structure, thermodynamic stability, and mutational robustness. The combined objective function is given by

where the neutrality is a number between 0 and 1, dG is the minimum free energy in kcal/mol, and distances are calculated using RNAdistance in the Vienna RNA package (supporting both the coarse-grain tree graphs that are called Shapiro representation, and the dot-brackets representation of the secondary structure).

The above objective function, as can be noticed in the third term, minimizes the distance between the desired shape and the mfe shape of the input. The distance is that as defined by tree edit distance over the Shapiro representation [13] (available in the Vienna RNA package by the routine 'b2shapiro') that provides the relaxation of the fine grain graph distance to a shape distance. The thermodynamic stability assessment distance is calculated here as the absolute value of the difference between the desired dG and the mfe dG of the input (minimum free energy in kcal/mol). The mutational robustness is evaluated by the neutrality. It is calculated as the absolute value of the difference between the observed neutrality of the input and the desired neutrality. Finally, in order to balance the result from being completely dominated by the shape consideration in the third term, a fourth term was added that minimizes the base-pair distance between the desired structure and the mfe structure of the input. Each one of the constraints receives its own weight as written in the formula above, and these weights can be further manipulated to emphasize various aspects of the minimization. Given the weights that have currently been fixed, we performed sensitivity analysis to determine the effect of different contributions by varying the input values and then examining how RNAexinv responds to these changes. Obviously, each run of RNAexinv produces a different answer because of the stochastic method employed. However, when changing each input value starting from a slight modification and continuing in an increasing manner, we have noticed that in addition to the expected change in the designed sequence, the output produced faithfully obeyed each time the constraints imposed. The shape remained exactly the same in all cases, having a high valued weight in the formula above, while the mutational robustness (neutrality) and thermodynamic stability (dG) were slightly changed from their desired values, by no more than 5% at most in the worst case. For brevity, avoiding redundancy, these results are not shown because as expected all input modifications produce a different sequence with a different predicted secondary structure, but the three constraints are fulfilled convincingly as a consequence of changing the input values each time.

RNAexinv utilizes a simulated annealing strategy to obtain the local minima, similar to the adaptive walk [5] and stochastic local search [12] strategies. Adaptive walk tests all the single-point mutation neighbors and takes the neighbor that has a better objective function to the next minimization step. Adaptive walk, however, has the tendency the get "stuck" in a local minimum if a sequence is best among its neighbors, although that minimum may not be the best solution even in that vicinity. Another widely applied strategy is stochastic local search. It combats the susceptibility of adaptive walk to getting stuck in a shallow local minimum by employing a constant probability to adapt a new sequence even if that sequence has a worse objective function result. As such, the stochastic local search strategy is able to escape shallow local minima. Simulated annealing also has a probability to adapt sequences with worse objective function results but that probability diminishes over the course of the minimization, and instead of looking only at the nearest neighbors a look ahead is now used to sample the vicinity of the sequence (the default look ahead is 4, not exhaustive, only sampling). Furthermore, to obtain a good sampling of the objective function landscape in consecutive runs, as mentioned before a random starting point is preferred over a static starting point to avoid repeatedly "falling into" the same local minimum.

To incorporate the physical measures listed above as constraints in the inverse RNA folding problem, the problem must first be relaxed from secondary structure to shape, as our previous simulations showed that such constraints can yield no solution [9]. Thus, instead of the RNA secondary structure, we used the simplified coarse-grained representation [13,14] as its shape. This is a convenient choice provided by the routine 'b2shapiro' in the Vienna RNA package. As a consequence, the inverse RNA folding problem becomes a reconstruction problem, given an RNA shape and physical attributes as constraints, to construct desired sequences. This will be demonstrated in the next section. The justification for relaxing the inverse RNA folding problem from that of an RNA secondary structure to an RNA shape emanates from the fact that if in the designed sequences there are a few more or less nucleotides in stems/loops but nevertheless these motif elements remain the same as in the initial input, such sequences are interesting to examine as candidate solutions to the design problem. An obvious advantage is in cases where there is a functional motif that should be strictly retained in the design procedure but further away from it there are non-functional motifs. In the non-functional motifs, an addition of one more or less nucleotide at the expense of another can well be justified if the overall designed sequence exhibits favourable properties in terms of stability and robustness.

### Availability

The package can be downloaded from http://www.cs.bgu.ac.il/~RNAexinv. After downloading, extract the file with the command:

> tar xvzf RNAexinvGUI.tar.gz

More details on how to run the program are contained in the ReadMe.html file that can be easily accessed from http://www.cs.bgu.ac.il/~RNAexinv.

### The package content

1. RNAexinv - performs RNAinverse to obtain a good initial sequence whose folding approximates the desired structure, followed by a simulated annealing heuristic with a four-nucleotide look ahead local search function to construct the desired output sequence for the extended inverse RNA folding problem. The RNAexinv routine predicts the secondary structure of RNA sequences using Vienna's RNAfold.

2. Java code - creates a "friendly" interface for the user. Receives as input an RNA secondary structure and relevant parameters for thermodynamic stability and mutational robustness, runs "RNAexinv", and generates the output that contains the designed sequences.

3. RNAfold - predicts minimum energy secondary structures and base pairing probabilities. The RNAfold program is taken from the Vienna RNA package.

4. RNAplot - draws the secondary structure of the RNA, given a sequence and the dot-bracket representation of the secondary structure.

### Preparation and Compilation

RNAexinv is currently available on a Linux platform. Therefore, all preparations and compilations should be performed on a Linux platform with Java and "GNU CC" compiler installed. RNAexinv has all its components already compiled and may be used without any compilations, but it has some components written in C that in some architectures may not work. In such a case, the Vienna RNA package should be downloaded from the web at http://www.tbi.univie.ac.at/~ivo/RNA/ and compiled. After the compilation finishes, files: "RNAplot" and "RNAfold" should be copied from Vienna RNA to the "RNAexinvGUI\bin" directory. All files that are already in this directory should be overwritten. In order to compile RNAexinv, please go to the RNAexinvGUI\RNAexinv directory and run the make command. Copy the created RNAexinv executable to the RNAexinvGUI\bin directory.

Make sure that all files in the "RNAexinvGUI\bin" directory are in an executable mode. If not, change their mode by typing the command: > chmod 700 file_name, where file_name is each file from the \bin directory.

## Results and discussion

**Figure 1:**
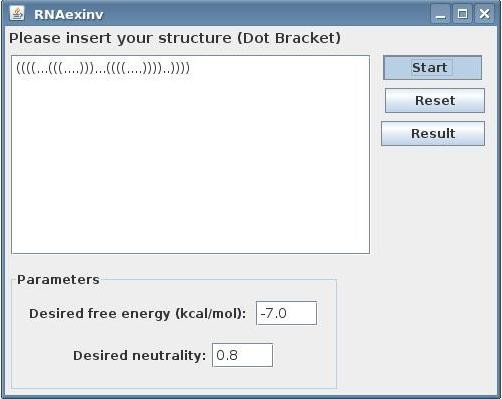
**RNAexinv Input Screen**. Initial GUI screen for providing the RNA secondary structure, as well as neutrality and minimum free energy values that the user would like to use for sequence design.

The input to the RNAexinv tool as shown in Figure 1 is simply an RNA secondary structure and two additional parameter values for thermodynamic stability (desired minimum free energy in kcal/mol) and mutational robustness (desired neutrality value from zero to one). Subsequently, after pressing the "Start" button, the RNAexinv program is executed several times and for each a designed sequence is constructed by the method described above. Basically, RNAexinv takes the structure, minimum free energy and neutrality values and constructs the RNA sequence that adheres to the three constraints most tightly (the first constraint being the RNA shape produced from the input secondary structure, while the second and third are thermodynamic stability and mutational robustness). Furthermore, the local search parameters can be customized to fit the particular needs of the run. There are three parameters inside the program that can be fine tuned in order to improve the local search fitness for a particular run:

1) The number of minimization steps (in the command line, RNAexinv -i <*number of steps *>). Increasing the number of steps will definitely increase CPU time but might increase the goodness of the results until a point when it will stride in the same local minimum.

2) The maximum distance of the look ahead (in the command line, RNAexinv -t <*neighbor distance *>). Increasing it will increase CPU time but might help the local search overcome shallow local minima.

3) Trying to tie the ends of the structure together (in the command line, RNAexinv -e).

Setting each parameter can influence the accuracy of RNAexinv result and the time to complete the run. Each user needs to balance the CPU time dedicated for each run and the number of repetitive runs. On the one hand, without enough CPU time for each run, the runs will not exploit their full potential. On the other hand, with too much CPU time for each run, the time will be wasted running in circles around the same minimum instead of starting from a different point to better examine the landscape.

**Figure 2:**
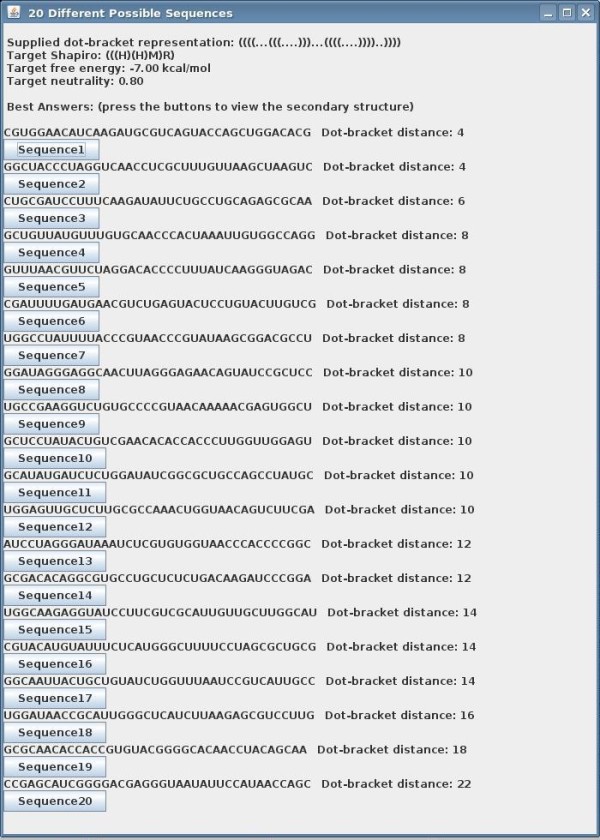
**List of Designed Sequences**. Main GUI screen for listing the designed sequences that are generated by multiple runs of RNAexinv. The scores on the right indicate the tree-edit distance between the predicted secondary structure of the designed sequence and that of the input.

**Figure 3:**
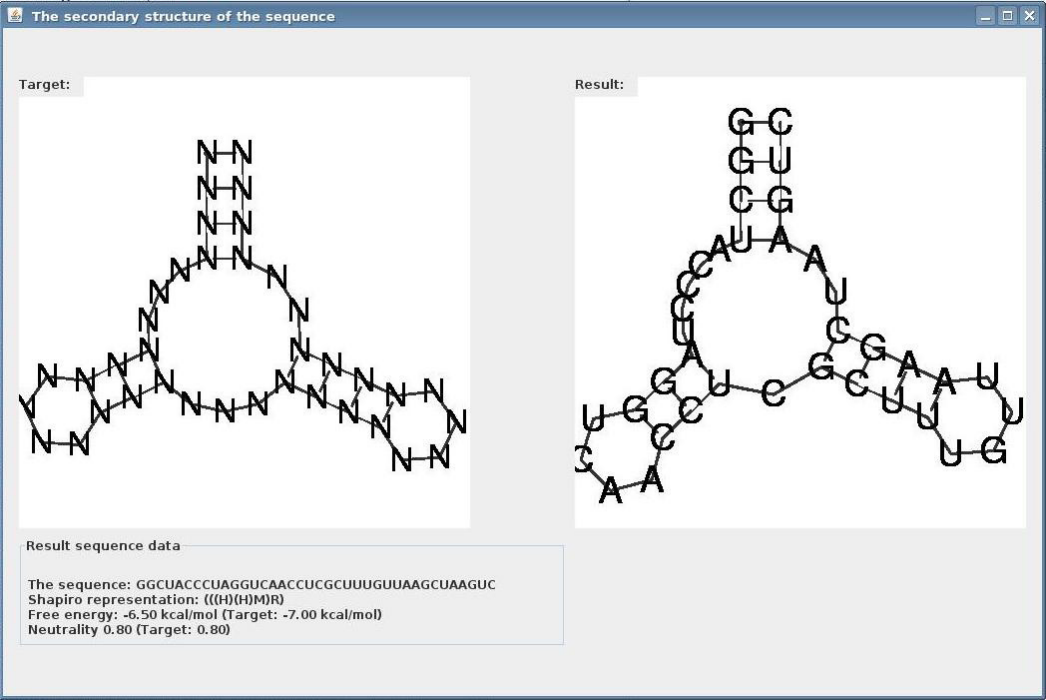
**Designed Sequence Data**. For each designed sequence, relevant secondary structure and accompanied information is provided along with a graphical drawing for both the given initial structure and the predicted secondary structure of the designed sequence.

The output of RNAexinv is demonstrated in Figures 2 and 3. Figure 2 illustrates the main window containing a list of designed sequences that are generated by multiple runs of the RNAexinv program. The scores on the right indicate the tree-edit distance between the predicted secondary structure of the designed sequence and that of the input. Next, the user can interactively view essential information by clicking on each of the designed sequences. Figure 3 is an illustration of the window provided for a particular designed sequence. Relevant secondary structure and accompanied information consists of a graphical drawing for both the given initial structure and the predicted secondary structure of the designed sequence, along with its computed minimum free energy and neutrality values.

**Figure 4:**
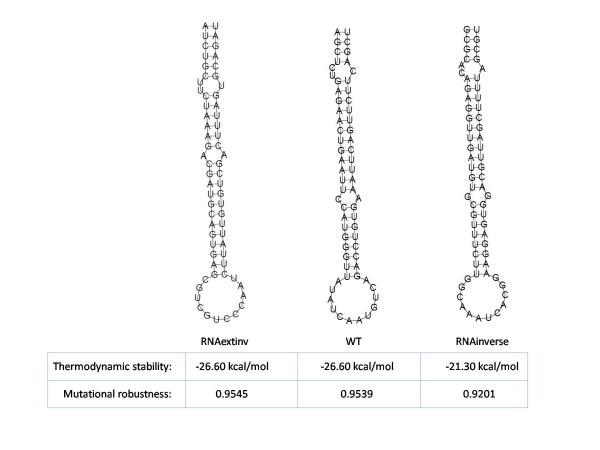
**Comparison of RNAexinv with RNAinverse for miR-146 Example**. Comparison of miR-146 wild-type structure (center) with the predicted structure of a designed sequence by RNAinverse (right) and the predicted structure of a designed sequence by RNAexinv (left). The physical attributes of the designed sequence by RNAexinv are significantly closer to that of the wild-type, while the same RNA shape as in the wild-type is retained.

We compared our suggested extension with the standard (non-extended) inverse RNA folding problem, in particular against RNAinverse [5], without loss of generality (the same conclusions also hold if we compare our method with RNA-SSD [6] or INFO-RNA [7]). The comparison depicted in Figure 4 was performed for an miR-146 example test case taken from [15], after it was verified in [9] by taking a collection of miRNAs from the microRNA registry [16] that miRNA precursors are significantly more thermodynamically stable and mutationally robust than random RNAs. Therefore, even though from an initial inspection of the structure drawings in Figure 4 it may appear that the RNAinverse result is in better agreement to the wild-type than the RNAexinv result, it should be noted that the values of the thermodynamic stability and mutational robustness of miR-146 are not taken into account when designing sequences with RNAinverse. In contrast, the sequences designed using RNAexinv possess highly similar values of thermodynamic stability and mutational robustness to that of the wild-type, potentially overweighing the better structural similarity achieved with RNAinverse for at least some problem specific applications (for example, in case the hairpin in Figure 4 is biologically meaningful but the exact location of the internal loops has no special significance). As for the computational effort, RNAinverse and INFO-RNA are considerably more efficient than RNAexinv, which is expected because the latter solves a much more demanding problem from the computational perspective. On a standard PC, the example in Figure 4 took 0.02 seconds using both RNAinverse and INFO-RNA, while it took 70 seconds for RNAexinv to calculate its result. However, the notable advantage of our more efficient method described and implemented here over the method proposed in [9] is that we managed to compute the extended inverse RNA folding in minutes on a standalone PC, for sequences in the length order of about 100 nt, instead of days on a parallel platform as previously attempted in [9]. Finally, in addition to the miRNA example shown in Figure 4, the P5abc subdomain example described in [9] was taken and for the relevant stochastic methods, the averages and standard deviations of the mutational robustness and thermodynamic stability were calculated over 1000 runs. The results are reported in Tables 1 &2. They provide further evidence for the benefit of RNAexinv when target (desired) values for mutational robustness and thermodynamic stability are given.

**Table 1:** Calculated Averages and Standard Deviations for dG and Neutrality for the miR-146 Example

Quantities Target/Program	Average of Neutrality	Standard Deviation of Neutrality	Average of dG (kcal/mol)	Standard Deviation of dG (kcal/mol)
Target	0.95	N/A	-26.60	N/A
RNAinverse	0.96	0.021	-29.03	5.14
RNAexinv	0.95	0.002	-26.56	0.20

Reported values of averages and standard deviations over 1000 runs with the methods compared, for mutational robustness (neutrality) and thermodynamic stability (dG in kcal/mol), performed on the miR-146 example shown in Figure 4.

**Table 2:** Calculated Averages and Standard Deviations for dG and Neutrality for the P5abc subdomain Example

Quantities Target/Program	Average of Neutrality	Standard Deviation of Neutrality	Average of dG (kcal/mol)	Standard Deviation of dG (kcal/mol)
Target	0.94	N/A	-25.60	N/A
RNAinverse	0.79	0.067	-15.42	3.21
RNAexinv	0.95	0.003	-25.01	1.49

Reported values of averages and standard deviations over 1000 runs with the methods compared, for mutational robustness (neutrality) and thermodynamic stability (dG in kcal/mol), performed on the P5abc subdomain example described in [9].

## Conclusions

In examining its biological relevance, RNAexinv can be used for designing sequences that adhere to the extended inverse RNA folding problem suggested in [9], but in practical CPU time. The implementation described here that is closer in type to previous related work for inverse RNA folding [4-7] is significantly more efficient than the parallel evolutionary computation implemented in [9]. From the illustrated example we can conclude that RNAexinv is able to design sequences that will not be taken into account by currently available programs for solving the inverse RNA folding problem, yet they will contain better features in terms thermodynamic stability and mutational robustness (and possibly other physical attributes, such as sequence complexity [17]) to mimic favorable properties of natural RNA sequences. RNAexinv successfully runs in concert with the Vienna RNA package that can be downloaded from http://www.tbi.univie.ac.at/~ivo/RNA and is freely available for download in http://www.cs.bgu.ac.il/~RNAexinv.

## Availability and requirements

**Project name: **RNAexinv

**Project home page: **http://www.cs.bgu.ac.il/~RNAexinv

**Operating system(s): **web access: not applicable, stand-alone: LINUX

Programming language: C, Java

**Other requirements: **stand alone:Java 1.4.0 or higher, GNU C compiler

**License: **None

**Any restrictions to use by non-academics: **None

## Competing interests

The authors declare that they have no competing interests.

## Authors' contributions

DB and AA conceived the study, coordinated and participated in software design and drafted the manuscript. AC worked on software design, carried out development and implementation, and participated in drafting the manuscript. All authors read and approved the final manuscript.
